# Evaluation of Selenium Concentrations in Patients with Crohn’s Disease and Ulcerative Colitis

**DOI:** 10.3390/biomedicines12102167

**Published:** 2024-09-24

**Authors:** Michał Chalcarz, Beniamin Oskar Grabarek, Tomasz Sirek, Agata Sirek, Piotr Ossowski, Mateusz Wilk, Katarzyna Król-Jatręga, Konrad Dziobek, Julia Gajdeczka, Jarosław Madowicz, Damian Strojny, Kacper Boroń, Jakub Żurawski

**Affiliations:** 1 Chalcarz Clinic, 60-567 Poznań, Poland; 2 Collegium Medicum, WSB University, 41-300 Dabrowa Górnicza, Poland; bgrabarek7@gmail.com (B.O.G.); drpiotrossowski@gmail.com (P.O.); mat1993wil@gmail.com (M.W.); katarzynakroljatrenga@gmail.com (K.K.-J.); konraddziobek28@gmail.com (K.D.); gajdeczkajulia@gmail.com (J.G.); jmadowicz@wsb.edu.pl (J.M.); strojny.ds@gmail.com (D.S.); 3 Department of Plastic Surgery, Faculty of Medicine, Academia of Silesia, 40-555 Katowice, Poland; drtstierka@gmail.com (T.S.); agatasirek85@gmail.com (A.S.); q375@icloud.com (K.B.); 4 Department of Plastic and Reconstructive Surgery, Hospital for Minimally Invasive and Reconstructive Surgery in Bielsko-Biała, 43-316 Bielsko-Biala, Poland; 5 Institute of Health Care, National Academy of Applied Sciences in Przemyśl, 37-700 Przemyśl, Poland; 6 New Medical Techniques Specjalist Hospital of St. Family in Rudna Mała, 36-054 Rudna Mala, Poland; 7 Department of Immunobiology, Poznan University of Medical Sciences, 60-567 Poznań, Poland; zurawski@ump.edu.pl

**Keywords:** Crohn’s disease, ulcerative colitis, inflammatory bowel disease, selenium, Crohn’s disease activity index, Harvey–Bradshaw index, simple endoscopic score for Crohn’s disease

## Abstract

Background/Objectives: In this study, serum selenium levels in patients with Crohn’s disease (CD) and ulcerative colitis (UC) were evaluated to identify potential predictive markers of disease activity. Conducted in 100 inflammatory bowel disease (IBD) patients (54 CD, 46 UC) and 100 healthy controls, this research provides novel insights through focusing on the regional selenium status of people with IBD in the Polish population, a demographic with limited existing data. Methods: Selenium concentrations were measured using inductively coupled plasma mass spectrometry (ICP-MS). Results: Significantly lower levels of selenium were observed in CD (64.79 µg/L ± 12.15 µg/L) and UC (68.61 µg/L ± 11.43 µg/L) patients when compared with the controls (90.52 ± 12.00 µg/L, *p* < 0.0001). Regression analysis identified leukocyte and erythrocyte counts and bilirubin as significant predictors of selenium levels in UC patients, while no significant predictors were found for CD. Conclusions: The findings suggest that selenium deficiency is linked to IBD and may serve as a non-invasive biomarker for disease severity, particularly in UC. This practical approach offers a potential alternative to invasive procedures such as endoscopy for monitoring disease progression. However, further research is needed to confirm these findings in larger populations and explore the therapeutic role of selenium supplementation in IBD management.

## 1. Introduction

Inflammatory bowel disease (IBD), which includes Crohn’s disease (CD) and ulcerative colitis (UC), is a chronic condition characterized by inflammation of the gastrointestinal tract [1,2]. The exact cause of IBD is not fully understood, but it is believed to result from a combination of genetic, environmental, and immunological factors [3,4,5]. This chronic inflammation leads to a range of symptoms, including abdominal pain, diarrhea, weight loss, and fatigue, significantly impacting the patient’s quality of life [6,7,8].

CD can affect any part of the gastrointestinal tract, from the mouth to the anus, and is characterized by transmural inflammation, which can lead to complications such as strictures, fistulas, and abscesses [9,10,11]. The symptoms of CD include abdominal pain, persistent diarrhea, weight loss, and fatigue [9,10,11]. The disease can have periods of remission and flare-ups, which vary in severity and frequency among patients [9,10,11].

On the other hand, UC primarily affects the colon and rectum and involves continuous mucosal inflammation, starting from the rectum and extending proximally [12,13,14,15]. UC is characterized by symptoms such as bloody diarrhea, urgency, abdominal cramping, and tenesmus [12,13,14,15]. Like CD, UC also has periods of remission and relapse, with symptom severity ranging from mild to severe [12,13,14,15]. 

Selenium, an essential trace element, plays a critical role in maintaining immune function and protecting cells from oxidative damage through its incorporation into selenoproteins. These proteins have various functions, including antioxidant defense and immune response regulation [16,17,18]. Selenium deficiency has been shown to exacerbate inflammatory conditions and has been associated with several chronic diseases, including IBD [19,20,21,22,23,24]. 

The novelty of this study lies in its focus on IBD patients in the Polish population, a demographic for which selenium concentration data are limited [25]. This regional focus can highlight potential geographical or dietary variations in selenium levels that may not have been studied elsewhere [26,27,28]. Understanding regional variations in selenium status and its relationship with IBD is crucial for developing tailored nutritional and therapeutic strategies [26,27,28]. Our study highlights the potential of using serum selenium levels as a non-invasive, cost-effective marker for monitoring IBD severity, reducing the reliance on invasive procedures such as endoscopy. This practical, patient-friendly approach makes our work highly valuable in clinical settings. Moreover, through evaluating the selenium concentration as a biomarker for predicting disease activity and severity, our research could lead to improved monitoring and enable personalized treatment, ultimately enhancing patient outcomes [29,30,31,32].

While Castro Aguilar-Tablada et al. examined selenium concentrations in patients with CD and UC and linked selenium deficiency to increased cardiovascular risk, our study provides novel insights by specifically focusing on the Polish population. In addition, our results illustrate the relationship between selenium levels and disease severity, which is quantified through clinical indices (Crohn’s Disease Activity Index, CDAI; Harvey–Bradshaw Index, HBI; and Clinical Activity Index, CAI). This adds a layer of precision to how selenium may be used to predict disease activity, which was not as explicitly explored in the study of Aguilar-Tablada et al. [33]. Additionally, Kudva et al. provided a general overview of selenium’s role in IBD but did not report detailed clinical correlations with disease severity [34]. Our research adds to the current knowledge through the use of selenium as a potential biomarker to predict IBD severity based on specific, validated clinical indices, such as the CDAI and CAI. Additionally, our use of inductively coupled plasma mass spectrometry (ICP-MS) to measure selenium concentrations increases technical accuracy, making the findings especially relevant for clinical application.

Stedman et al. focused on selenium supplementation for patients with ulcerative colitis, but they did not comprehensively investigate the association between selenium levels and disease activity. In contrast, our study goes beyond supplementation by thoroughly analyzing selenium concentration as a predictive marker for disease severity in both CD and UC [35]. Furthermore, our paper presents a modern approach to selenium measurement and a regional focus on a population with limited data, which provide new insights into the geographic variability in selenium.

Therefore, the aim of this study is to assess the feasibility of using serum selenium concentration changes to predict the occurrence and evaluate the severity of IBD in patients with Crohn’s disease and ulcerative colitis.

## 2. Materials and Methods

### 2.1. Ethics

This study was conducted in accordance with the Helsinki Declaration guidelines and received approval from the Bioethics Committee of the Medical University of Karol Marcinkowski in Poznań (approval no. 39/13; 17 December 2021). Data confidentiality and patient anonymity were rigorously maintained throughout the study. All identifying information was removed before database analysis, ensuring that individual patients cannot be identified from this article or the database. Written informed consent was obtained from all participants.

### 2.2. Characteristics of the Study and Control Groups

All patients received treatment at the Division of Gastroenterology within the General, Oncological, and Colorectal Surgery Department of the Multispecialty Municipal Hospital named after Józef Struś in Poznań, as well as the Stoma Clinic affiliated with the Department of General and Endocrine Surgery at Clinical Hospital No. 2 in Poznań. The baseline characteristics of healthy controls were matched with those of patients with UC and CD. All patients had a confirmed diagnosis based on radiological, histological, and clinical criteria. No patients or healthy volunteers received Se supplementation. All colonoscopies were performed by a single gastroenterologist, who also graded the endoscopic scores. The gastroenterologist was blinded to the selenium levels.

### 2.3. Assessment of CD Activity

CD activity was assessed using both clinical and biochemical data. Disease activity was categorized into four levels based on endoscopic scores (the Simple Endoscopic Score for Crohn’s Disease, SES-CD) and clinical scores CDAI, and HBI, defined as follows: remission (SES-CD ≤ 2, CDAI < 150, HBI ≤ 4), mild activity (SES-CD 3–6, CDAI 150–220, HBI 5–7), moderate activity (SES-CD 7–15, CDAI 221–450, HBI 8–16), and severe activity (SES-CD > 15, CDAI > 450, HBI > 16) [36].

### 2.4. Assessment of UC Activity

The CAI for ulcerative colitis, also known as the Rachmilewitz Index, measures disease severity based on specific clinical parameters. These parameters include the number of stools per day, the presence of blood in the stools, general well-being, abdominal pain, extraintestinal manifestations, and body temperature. Each parameter is scored, and the total CAI score is the sum of these points. For the number of stools per day, the scoring ranges from 0 points for 0–1 stool to 3 points for more than 6 stools. Blood in stools is scored from 0 points for no blood to 3 points for mostly blood. General well-being is rated from 0 points for feeling very well to 4 points for feeling terrible. Abdominal pain is scored from 0 points for none to 3 points for severe pain. Extraintestinal manifestations are assigned 0 points for none, 1 point for one manifestation, and 2 points for two or more manifestations. Body temperature is scored from 0 points for temperatures ≤37.0 °C to 3 points for temperatures >39.0 °C. 

The total CAI score is the sum of the points for each parameter. Generally, CAI scores of 0–4, 5–10, 11–15, and 16–21 indicate remission or very mild disease, mild disease activity, moderate disease activity, and severe disease activity, respectively. This scoring system assists healthcare providers in monitoring disease activity in patients with ulcerative colitis and making informed treatment decisions [37]. 

Blood samples were collected from patients after obtaining informed consent, and the serum was separated immediately by centrifugation and stored at −80 °C until analysis.

### 2.5. Venous Blood Collection from Patients with IBD and Healthy Volunteers

Venous blood was collected using two types of vacuum blood collection tubes: with an anticoagulant (potassium Ethylenediaminetetraacetic acid(EDTA)) and without an anticoagulant. In the tube with the anticoagulant, 2.7 cm^3^ of blood was collected for same-day measurements of leukocytes, erythrocytes, and hemoglobin. Two tubes without the anticoagulant were used to collect 6 cm^3^ of blood for selenium measurements and 4.9 cm^3^ of blood for other biochemical analyses. After coagulation, the blood in both tubes was centrifuged at 1300× *g* for 10 min at room temperature. The obtained serum was pipetted into two new polypropylene tubes.

### 2.6. Hematological and Biochemical Analyses

From the serum collected in the first tube, same-day measurements of C-reactive protein (CRP), bilirubin, alanine aminotransferase (ALT), and aspartate aminotransferase (AST) concentrations were performed. The leukocyte, erythrocyte, and hemoglobin contents were measured using a Sysmex KX-21N analyzer (Bioprom, Thessaloniki, Greece). The concentrations of CRP, bilirubin, ALT, and AST in the serum were measured using a Cobas c 501 automatic biochemical analyzer (Roche Polska sp. z o.o., Warsaw, Poland). 

### 2.7. Assessment of Selenium Levels in Serum from IBD Patient and Control Groups

The selenium concentrations in serum samples were determined using inductively coupled plasma mass spectrometry (ICP-MS NexION 350D, Perkin Elmer, Waltham, MA, USA), with methane employed to mitigate polyatomic interference. Calibration standards were created by diluting 10 mg/L Multi-Element Calibration Standard 3 (PerkinElmer Pure Plus, PerkinElmer Life and Analytical Sciences, Waltham, MA, USA) using a solution of 0.65% nitric acid (Suprapur, Merck, Germany) and 0.002% Triton X-100 (PerkinElmer, Tokyo, Japan). Calibration curves were established using four different concentrations: 0.1 g/L, 0.5 g/L, 1 g/L, and 2 g/L. Germanium (PerkinElmer Pure, PerkinElmer Life and Analytical Sciences, Waltham, MA, USA) served as the internal standard, while ClinChek Plasma Control Level I (Recipe, Germany) was used as the reference material [38].

Reference selenium concentration values accounting for sex differences were sourced from the “Innovative Medicine” diagnostic laboratory affiliated with READ-GENE S.A. in Szczecin, Poland. The reference ranges were 75–85 μg/L for women and 85–115 μg/L for men. Each biological test was performed with three technical replicates.

### 2.8. Statistical Analysis

Statistical analyses were conducted using Statplus software (AnalystSoft, Brandon, FL 33511, USA, https://www.analystsoft.com/en/products/statplusmacle/), with the significance level (*p*) set below 0.05. First, the conformity of numerical data to the normal distribution was assessed using the Shapiro–Wilk test, which confirmed the null hypothesis. Consequently, further analyses were performed using parametric tests, specifically a one-way analysis of variance (ANOVA). Scheffe’s post hoc test was used for subsequent comparisons. Additionally, before performing the ANOVA, the homogeneity of variances was verified using Levene’s test. Data are presented as means ± standard deviations and 95% confidence intervals (95% confidence intervals (CIs)). The Bonferroni correction was applied to adjust for multiple comparisons.

## 3. Results

### 3.1. Results of Subjects’ Characteristics

The clinical characteristics of patients with IBD and healthy volunteers are presented in Table 1.

### 3.2. Comparison of Hematological and Biochemical Parameters between Patients with IBD and Healthy Volunteers

First, we evaluated differences in the selected hematological and biochemical parameters between the group of patients with CD or UC and the control group. Statistical analysis revealed statistically significant differences in the levels of erythrocytes, hemoglobin, ALT, and AST between the groups (one-way ANOVA; *p* < 0.05). Next, Scheffe’s post hoc test was conducted for these parameters to determine the groups (patients and healthy volunteers) between which the assessed values differed significantly. The results are presented in Table 2.

### 3.3. Evaluation of Selenium Concentrations in the Study and Control Groups

**Figure 1:**
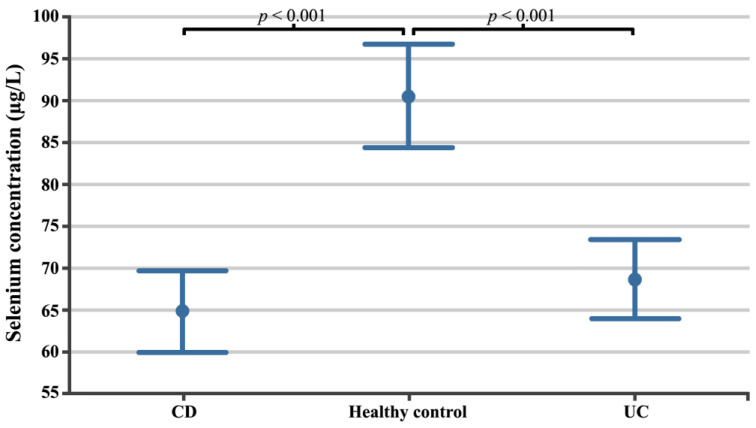
Selenium concentrations in patients with CD, patients with UC, and healthy volunteers. CD, Crohn’s disease; UC, ulcerative colitis.

The selenium concentration in the CD patient group was 64.79 µg/L ± 12.15 µg/L (95% confidence intervals (CI) 59.88 µg/L 9l.69 µg/L; min 36.43 µg/L, max. 91.35 µg/L) and was lower than in UC patients (68.61 µg/L ± 11.43 µg/L; 95%CI 63.89 µg/L–73.32 µg/L; min. 43.74 µg/L; max. 88.76 µg/L), while in the healthy volunteer group it was 90.52 µg/L ± 12.00 µg/L (95%CI 84.35 µg/L–96.69 µg/L; min. 76.10 µg/L; max. 119.12; µg/L *p* < 0.0001). Scheffe’s post hoc test showed statistically significant differences between healthy volunteers and both the CD (*p* < 0.0001) and UC (*p* < 0.0001) patient groups. The differences in selenium concentrations between the evaluated groups are shown in Figure 1. 

### 3.4. Selenium Concentrations in Patients with CD Depending on the Severity of the Disease

In this study, 29 patients with CD had high disease activity, with a CD activity index (CDAI) greater than 150, while 25 patients exhibited low disease activity, with a CD activity index less than 150 [39]. 

**Figure 2:**
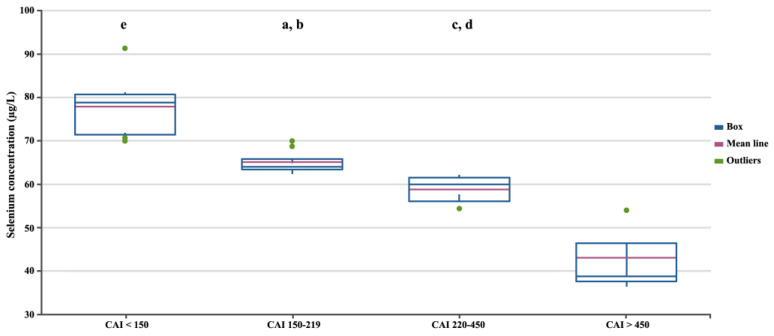
Selenium concentrations in patients with CD depending on the CDAI. Statistically significant differences: a, CDAI 150–219 vs. CDAI < 150; b, CDAI 150–219 vs. CDAI > 450; c, CDAI 220–450 vs. CDAI < 150; d, CDAI 220–450 vs. CDAI > 450; e, CDAI < 150 vs. CDAI > 450.

**Figure 3:**
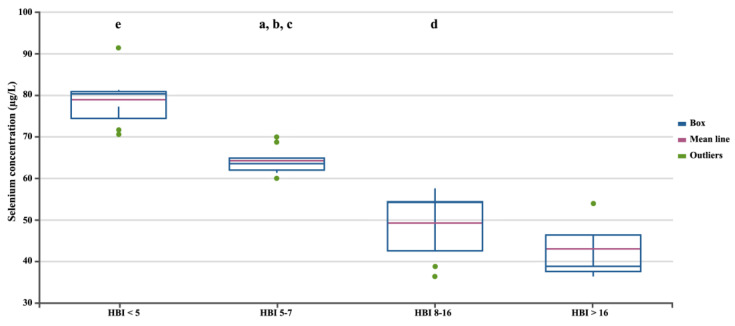
Selenium concentrations in patients with CD depending on the HBI. Statistically significant differences: a, HBI 5–7 vs. HBI 8–16; b, HBI 5–7 vs. HBI < 5; c, HBI 5–7 vs. HBI > 16; d, HBI 8–16 vs. HBI < 5; e, HBI < 5 vs. HBI > 16.

**Figure 4:**
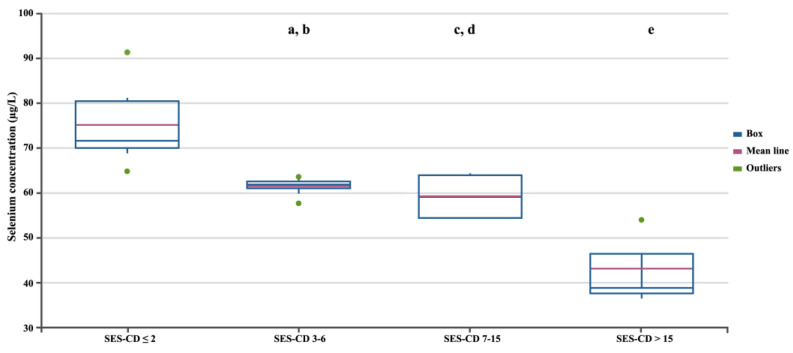
Selenium concentrations in patients with CD depending on the SES-CD. Statistically significant differences: a, SES-CD 3–6 vs. SES-CD > 15; b, SES-CD 3–6 vs. SES-CD ≤ 2; c, SES-CD 7–15 vs. SES-CD > 15; d, SES-CD 7–15 vs. SES-CD ≤ 2; e, SES-CD > 15 vs. SES-CD ≤ 2.

In the next stage, we assessed differences in selenium concentration in patients with CD depending on the severity of the disease, measured using three independent factors (Figure 2, Figure 3 and Figure 4). Regardless of the criteria adopted for disease severity, the increase in disease severity was associated with a decrease in the serum selenium concentration in CD patients (*p* < 0.05). Figure 2, Figure 3 and Figure 4 show the differences in the selenium concentration in patients with CD depending on the CDAI, HBI, and SES-CD indices, respectively. 

### 3.5. Selenium Concentrations in Patients with UC Depending on Disease Severity 

**Figure 5:**
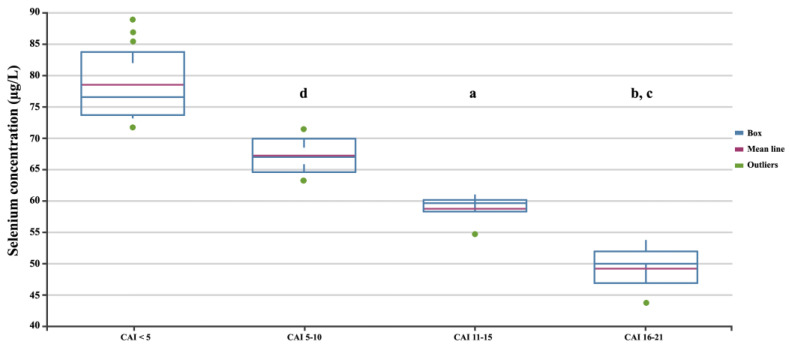
Selenium concentrations in patients with UC depending on the CAI. Statistically significant differences: a, SES-CD 3–6 vs. SES-CD > 15; b, SES-CD 3–6 vs. SES-CD ≤ 2; c, SES-CD 7–15 vs. SES-CD > 15; d, SES-CD 7–15 vs. SES-CD ≤ 2.

Among patients with UC, 21 had high disease activity, with a Clinical Activity Index greater than 5, as defined by Lichtiger et al. [37], and were considered to be in the active phase. Additionally, 25 UC patients had low disease activity. We further assessed differences in selenium concentration according to the disease severity score based on the CAI scale (Figure 5). Statistical analysis showed that selenium concentrations statistically significantly decreased with disease severity ANOVA; *p* < 0.05).

### 3.6. Multivariate Stepwise Linear Regression Analysis of Serum Selenium Levels in CD Patients

The overall model explained 64% of the variance in serum selenium levels (R-squared (R^2^) = 0.64), but it was not statistically significant (F-statistic (F) = 1.38, *p* = 0.33). None of the variables reached statistical significance independently. The leukocyte count was negatively associated with selenium levels (regression coefficient (B) = −2.67988, *p* = 0.20832), but not significantly. Similarly, the erythrocyte count, hemoglobin levels, CRP, bilirubin, ALT, AST, CDAI, HBI, and SES-CD did not significantly contribute to the model (Table 3).

### 3.7. Multivariate Stepwise Linear Regression Analysis of Serum Selenium Level in UC Patients

The overall model explained 83% of the variance in serum selenium levels (R^2^ = 0.83) and was statistically significant (F = 6.24, *p* = 0.005). Among the variables, the leukocyte count (B = −2.39850, *p* = 0.00015), erythrocyte count (B = −2.61948, *p* = 0.02747), and bilirubin (B = 0.48544, *p* = 0.01480) were significant predictors of serum selenium levels. The negative associations with leukocyte and erythrocyte counts suggest that higher levels of these parameters are associated with lower selenium levels. In contrast, bilirubin had a positive association with selenium levels. Hemoglobin, CRP, ALT, AST, and CAI did not significantly predict selenium levels in this analysis (Table 4).

## 4. Discussion

Effectively managing intestinal inflammation is essential for achieving the treat-to-target approach in CD and UC, necessitating precise and reliable assessment of disease severity. The current methods for evaluating disease activity include clinical symptoms, colonoscopy, gastroscopy, radiological imaging of the small intestine, and biopsies from multiple gastrointestinal segments to ensure a thorough histologic evaluation and exclude chronic infectious intestinal diseases [36,40]. However, the high costs, complexity, invasiveness, and limited availability of endoscopic procedures restrict frequent monitoring [41,42]. Moreover, indices such as the CDAI, CAI, and HBI, which are based on patient-reported symptoms, can lead to inaccuracies due to variations in symptom perception and reporting. Consequently, there is a critical need for non-invasive and objective biomarkers to accurately assess disease activity in CD and UC. 

IBD has been associated with an increased risk of colon cancer [43]. While Se and selenoproteins are known for their antioxidant functions and their significance to the cardiovascular system, in addition to the recognized role of Se-enriched diets in reducing colon cancer risk, there are limited data on the influence of Se on IBD [44,45]. Se supplementation has shown protective effects against tissue damage in chemically induced UC by altering the expression of genes related to mitochondria-regulated cell death [46]. Epidemiological studies indicate lower Se concentrations in patients with CD or UC, which is supported by clinical findings of reduced blood Se levels in IBD patients [47,48,49,50]. Huang et al. identified selenium as a vital regulator of T-cell responses in CD, noting that dietary Se supplementation alleviates these symptoms by polarizing macrophages from an M1- to an M2-like phenotype, thereby promoting gut epithelial damage resolution [51,52]. Furthermore, adequate Se levels can modify the arachidonic acid pathway, leading to the increased production of anti-inflammatory mediators and reduced inflammation [53].

In this study, we measured selenium levels in patients with CD or UC and healthy volunteers. The serum selenium concentration was lower in CD patients than in UC patients, although these differences were not statistically significant (*p* > 0.05), suggesting that selenium concentrations cannot be used to differentiate between CD and UC cases. Aguilar-Tablada et al. also observed significantly lower serum selenium levels in patients with CD compared to those with UC, which is consistent with our findings [33]. However, further analysis revealed that selenium levels significantly decreased with the severity of disease symptoms in both CD and UC patients (*p* < 0.05).

Of particular note are our multiple regression analysis results. In CD patients, we found that selenium levels were strongly associated with hemoglobin and C-reactive protein (CRP) levels, as well as with the CDAI and SES-CD scales, although these associations were not statistically significant (*p* > 0.05). The SES-CD is considered a relatively reliable tool for assessing the severity of CD. In this study, the correlation between SES-CD scores and serum selenium levels was strong at all levels of disease severity. The associations between the four SES-CD categories and serum selenium concentrations suggest that serum selenium status is a vital indicator of intestinal mucosal inflammation in patients with CD. Furthermore, the cost of a serum selenium assay is approximately 1% of that of an endoscopy, with results available within two days, making the serum selenium assay significantly more cost-effective than endoscopy.

The CDAI and HBI are traditional scoring methods for evaluating the activity of CD based on clinical symptom severity [54,55]. In our study, we observed significant inverse relationships between the serum selenium concentration and the severity scores of both the CDAI and HBI in univariate analyses (*p* < 0.05). This correlation is consistent with findings from other research on selenium’s role in colitis [56,57]. The serum selenium concentration could potentially serve as an additional indicator, combined with the CDAI and HBI scores. However, these scores are inherently subjective and can be influenced by factors such as the patient’s tolerance to long-term symptoms. Therefore, they should be interpreted in conjunction with other diagnostic tools. The subjectivity of the CDAI and HBI likely contributed to the lack of significant associations between serum selenium levels and these indices in our multivariate analysis (*p* > 0.05).

In contrast, for patients with UC, regression analysis showed that selenium levels are most strongly related to the leukocyte and erythrocyte counts and bilirubin concentration (*p* < 0.05). Statistical analysis revealed a relationship between selenium levels and the CAI, but it was not statistically significant (*p* > 0.05).

Furthermore, we found significantly lower erythrocyte and hemoglobin concentrations accompanied by active inflammation (based on the CRP result) in patients with CD and UC, which also contributes to the reduced selenium concentrations in the study group. The decreased selenium levels observed are probably partly related to an insufficient supply of macro- and micro-elements from the diet in patients with CD or UC, leading to malnutrition [58,59]. Yan et al. conducted a study to investigate the correlation between serum selenium levels and disease activity in patients with CD. The study, which included 135 participants, revealed an inverse correlation between the serum selenium concentration and disease severity, suggesting that selenium might be a valuable marker for monitoring disease activity in CD alongside other factors [60]. Some researchers have proposed that selenium supplementation could enhance the efficacy of probiotics, potentially reducing the inflammation associated with CD [61,62]. Additionally, Keshteli et al. found that a diet rich in anti-inflammatory ingredients could modify the gut microbiota composition in patients with UC, leading to metabolic changes that help maintain clinical remission [63]. Maintaining adequate selenium levels is also associated with a lower risk of cardiovascular disease in patients with IBD [33]. Furthermore, Short et al. highlighted the crucial role of selenoprotein P (SEPP1) in regulating intestinal homeostasis, which affects inflammation and the risk of colorectal cancer [64]. 

These findings significantly contribute to the understanding of selenium’s role in inflammatory bowel disease (IBD) and its potential as a biomarker for disease activity. 

## 5. Conclusions

This study highlighted a significant reduction in serum selenium levels in patients with CD and UC when compared with healthy controls, underscoring the potential role of selenium in the pathophysiology of IBD. The results suggest that selenium deficiency may be linked to higher disease activity, particularly in UC, where the leukocyte and erythrocyte counts and bilirubin levels were significant predictors of selenium concentrations. While selenium levels alone may not differentiate between CD and UC, they offer valuable supplementary information for assessing disease severity. Importantly, the findings suggest that the serum selenium concentration could serve as a cost-effective, non-invasive biomarker for monitoring IBD severity, reducing the need for more invasive diagnostic methods such as endoscopy.

This study also demonstrates the potential utility of using selenium levels in personalized treatment strategies, as maintaining adequate selenium levels could mitigate disease severity and improve patient outcomes. Given the growing interest in nutritional approaches to managing chronic diseases, further research should focus on confirming these findings in larger, more diverse populations, exploring the underlying mechanisms of selenium’s role in IBD, and evaluating the effectiveness of selenium supplementation as part of a broader therapeutic approach. This study opens avenues for future studies aimed at refining the management of IBD and improving the quality of life of patients suffering from these debilitating diseases.

## Figures and Tables

**Table 1:** Clinical characteristics of patients with IBD and healthy volunteers.

	CD (*n* = 54)	Control (*n* = 100)	UC (*n* = 46)
Men/women	31/23	52/48	25/21
Age (years)	41.87 ± 8.91	41.17 ± 6.71	42.16 ± 4.81
Disease duration (years)	9.81 ± 1.23	-	8.12 ± 2.01
BMI (kg/m^2^)	21.09 ± 2.65	24.56 ± 2.19	22.16 ± 2.18
Type of CD			
Ileal	9	-	-
Ileocolonic	39	-	-
Colonic	6	-	-
CDAI < 150	25	-	-
CDAI 150–219	16	-	-
CDAI 220–450	10	-	-
CDAI > 450	3	-	-
HBI < 5	24	-	-
HBI 5–7	19	-	-
HBI 8–16	11	-	-
HBI > 16	3	-	-
SES-CD ≤ 2	23	-	-
SES-CD 3–6	26	-	-
SES-CD 7–15	3	-	-
SES-CD > 15	2	-	-
Type of UC			
Proctitis	-	-	37
Left-side colitis	-	-	5
Pancolitis	-	-	4
CAI < 5	-	-	25
CAI 5–10	-	-	13
CAI 11–15	-	-	6
CAI 16–21	-	-	2

CAI, Clinical Activity Index for ulcerative colitis; CD, Crohn’s disease; CDAI, Crohn’s Disease Activity Index; UC, ulcerative colitis; HBI, Harvey–Bradshaw Index; SES-CD, Simple Endoscopic Score for Crohn’s Disease; BMI, Body Mass Index.

**Table 2:** Comparison of hematological and biochemical parameters between patients with IBD and healthy volunteers.

Parameters	Reference Range	CD (*n* = 54)	Control (*n* = 100)	UC (*n* = 46)	*p*-Value (ANOVA)
Leukocytes (tys/μL)	4.23–9.07	7.73 ± 2.59 [6.68–8.79]	7.02 ± 1.75 [5.91–8.14]	7.48 ± 2.38 [6.49–8.46]	0.69
Erythrocytes (mln/µL)	4.63–6.06	4.32 ± 0.68 [4.05–4.60]	5.16 ± 0.77 [4.66–5.64]	4.45 ± 0.76 [4.13–4.76]	0.006 ^1,2^
Hemoglobin (mmol/L)	13.7–17.5	7.54 ± 0.99 [7.14–7.94]	14.55 ± 1.02 [13.90–15.20]	7.88 ± 0.93 [7.50–8.27]	<0.0001 ^2^
CRP (mg/L)	0–5	26.44 ± 37.09 [11.46–41.52]	2.01 ± 0.90 [1.44–2.58]	25.47 ± 52.30 [2.85–48.09]	0.19
Bilirubin (µmol/L)	3.40–20.50	10.43 ± 14.56 [3.62–17.24]	12.38 ± 4.13 [9.75–15.00]	10.20 ± 9.10 [6.05–14.34]	0.84
ALT (U/I)	0–45	15.20 ± 10.70 [10.78–19.62]	12.83 ± 3.61 [10.53–15.13]	21.85 ± 11.11 [16.65–27.05]	0.03 ^2^
AST (U/I)	11–34	15.96 ± 4.71 [13.97–17.95]	14.58 ± 2.35 [13.09–16.08]	21.35 ± 9.034 [17.12–25.58]	0.006 ^2,3^

Data are presented as mean ± standard deviation [95% confidential interval]. *p*-value, probability value, CD, Crohn’s disease; UC, ulcerative colitis; CRP, C-reactive protein; ALT, alanine aminotransferase; AST, aspartate aminotransferase. ANOVA, one-way analysis of variance, Statistical differences (*p* < 0.05 according to Scheffe’s post hoc test): ^1^ CD vs. control; ^2^ UC vs. control; ^3^ UC vs. CD.

**Table 3:** Multivariate stepwise linear regression analysis of factors associated with serum selenium levels in patients with Crohn’s disease.

	B	SEM	β	t	*p*
Leukocytes (tys/μL)	−2.680	1.958	−0.495	−1.369	0.208
Erythrocytes (mln/µL)	−2.801	13.746	−0.115	−0.204	0.844
Hemoglobin (mmol/L)	11.286	7.636	0.870	1.478	0.178
CRP (mg/L)	−0.241	0.130	−0.702	−1.847	0.102
Bilirubin (µmol/L)	−0.337	0.835	−0.108	−0.403	0.698
ALT (U/I)	0.635	0.826	0.332	0.769	0.464
AST (U/I)	−0.416	1.346	−0.148	−0.309	0.765
CDAI	0.542	0.414	0.510	1.311	0.226
HBI	0.300	0.373	0.295	0.805	0.444
SES-CD	−1.044	0.591	−1.017	−1.766	0.115

R = 0.8; R^2^ = 0.64; F = 1.38; *p*= 0.33; B, regression coefficient; SEM, standard error of the mean; β, standardized coefficient, t, t-statistic.

**Table 4:** Multivariate stepwise linear regression analysis of factors associated with serum selenium level in patients with UC.

	B	SEM	β	t	*p*
Leukocytes (tys/μL)	−2.399	21.439	−0.538	5.921	0.000
Erythrocytes (mln/µL)	−2.620	0.930	−0.140	−2.579	0.027
Hemoglobin (mmol/L)	−0.4798	4.070	−0.041	−0.644	0.534
CRP (mg/L)	−0.105	2.143	−0.511	−0.224	0.827
Bilirubin (µmol/L)	0.485	0.036	0.396	−2.939	0.015
ALT (U/I)	0.467	0.296	0.453	1.642	0.132
AST (U/I)	−0.278	0.370	−0.220	1.263	0.235
CAI	−0.456	0.400	−0.437	−0.694	0.503

R = 0.91; R^2^ = 0.83; F = 6.24; *p* = 0.005; B, regression coefficient; SEM, standard error of the mean; β, standardized coefficient; CAI, Clinical Activity Index for ulcerative colitis, R, correlation coefficient.

## Data Availability

The data used to support this study’s findings are included in this article. The data will not be shared due to third-party rights and commercial confidentiality.
